# Doing New Research? Don't Forget the Old

**DOI:** 10.1371/journal.pmed.0010035

**Published:** 2004-11-30

**Authors:** Mike Clarke

## Abstract

Nobody should do a new research study, says Clarke, without first systematically reviewing the literature. And journal editors should insist that all research papers are accompanied by an up-to-date systematic review

## Teaser

Nobody should do a trial without reviewing what is known

On May 2, 1898, George Gould used his address to the founding meeting of the Association of Medical Librarians in Philadelphia to present a vision of the future of health information. ‘I look forward,’ he said, ‘to such an organisation of the literary records of medicine that a puzzled worker in any part of the civilised world shall in an hour be able to gain a knowledge pertaining to a subject of the experience of every other man in the world’ [1]. Has his vision been realised?

## Information Overload

In these early years of the 21st century, with tens of thousands, if not hundreds of thousands, of new research articles being published every year, people who need to make decisions about health care are much more overwhelmed with information than they were in 1898. Some of this information is of good quality, but some of it is not. Thus, anyone wishing to use the health literature to make well-informed decisions must both identify the relevant research from amidst this vast amount of information and then appraise it. This is an impossible task for many. Even though making access to the literature easier and cheaper will increase the ability of people to find research, it will also reveal just how much information there is out there and how daunting is the task of making sense of it.

You can get a good idea of the size of the task—and of how electronic publishing and the Internet have transformed the situation—by imagining the following four steps. How long would it take to find articles in your area of interest by paging through back copies of a relevant journal? What about by using its index? Now imagine finding articles of interest to you by going to the Internet and searching PubMed (www.ncbi.nlm.nih.gov/PubMed). What about if you searched the whole of the Internet with one or more search engines? Almost certainly, as the speed of the search increased through these four approaches, so would the number of articles retrieved—and also the time that it would take to read through them, appraise them, and decide if they were relevant to whatever decision you were trying to make.[Fig pmed-0010035-g001]

**Figure pmed-0010035-g001:**
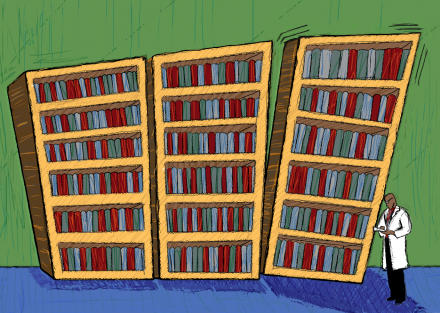
Doctors are overloaded with information, much of it irrelevant to their practice (Illustration: Rusty Howson, sososo design)

Should you just take a shortcut by relying on a single study in a high-profile journal? No. Sampling in this way might lead you to research whose findings are the most striking or atypical, but you would miss similar research that was ‘less fortunate’ with its results. However good the conduct of a piece of research, chance effects mean that some studies will produce an overestimate and some an underestimate of the true effects, but the easy-to-find literature is likely to be dominated by the former [2]. In addition, using a sample rather than the whole body of relevant research will have less statistical and evidential power to answer the question of interest.

## The Value of Systematic Reviews

Systematic reviews provide a means to minimise these problems. In systematic reviews, the methods to be followed are stated and an attempt is made to identify, appraise, and where appropriate, statistically combine, all relevant research. In fact, a decade before George Gould's address, Lord Rayleigh, at the meeting of the British Association for the Advancement of Science in Montreal, had described such a process for scientific research in general.

‘If, as is sometimes supposed,’ he said, ‘science consisted in nothing but the laborious accumulation of facts, it would soon come to a standstill, crushed, as it were, under its own weight. The suggestion of a new idea, or the detection of a law, supersedes much that has previously been a burden on the memory, and by introducing order and coherence facilitates the retention of the remainder in an available form. Two processes are thus at work side by side, the reception of new material and the digestion and assimilation of the old. One remark, however, should be made. The work which deserves, but I am afraid does not always receive, the most credit is that in which discovery and explanation go hand in hand, in which not only are new facts presented, but their relation to old ones is pointed out’ [3].

## Relating the New to the Old

If today's health researchers discussed their findings in the context of relevant, already-existing research, many of the problems of information overload would be eased. You would only need to find the most recent report of a relevant study; its discussion section would place that study within the context of an updated systematic review. This was suggested by the original CONSORT statement on the reporting of randomised trials in 1996 [4]. However, studies of five of the major general medical journals (*Annals of Internal Medicine, BMJ, Lancet, JAMA,* and the *New England Journal of Medicine*) in 1997 and 2001 found that this was not the case, at least for these journals. Only two of more than 50 reports of randomised trials in these journals in May of those two years included an updated systematic review [5,6].

Including an updated systematic review along with a report of a randomised trial (or any other piece of research) might seem too much to expect of researchers, who might not feel able or willing to do the additional work required. However, the absence of a review should raise the question: on what did the researchers base the design of their new study? To embark on a new study without first systematically reviewing what has been done before is to risk doing research for which the answer is already known. It would also mean that the researchers had denied themselves the opportunity to learn from the successes and failures of others when designing their own study. In addition, researchers have a responsibility to the participants in their research to make sure that the study is of the most appropriate design possible. To help make sure that this is the case, when designing a new study researchers should ensure that they have been adequately informed about what research has been done previously. Box 1 lists practical suggestions to researchers for making sure that their new study builds on prior knowledge.

**Box 1. Practical Suggestions for Researchers**

- Conduct a systematic review of your research question before embarking on a new study, or identify a relevant review done by someone else.
- Design your study to take account of the relevant successes and failures of the prior studies, and of the evidence within them.
- Discuss the findings of your study in the context of an updated systematic review of relevant research.
- Publish the systematic review within, alongside, or shortly after the report of your study.
- Provide information from your study to others doing systematic reviews of similar topics.

It might even be the case that the researchers are able to draw on the work of others, who already have done a systematic review of the relevant topic. Over the last decade, The Cochrane Collaboration (Box 2) has produced more than 2,000 Cochrane systematic reviews (www.cochrane.org) [7]. There are thousands more reviews scattered throughout the literature (www.york.ac.uk/inst/crd/darefaq.htm). And with the ability to publish longer versions of articles on the Internet than are practical in print, concerns about article length should no longer be a barrier to the inclusion of a systematic review.

**Box 2. The Cochrane Collaboration**

The Cochrane Collaboration (www.cochrane.org) is an international, nonprofit, and independent organisation dedicated to helping people make well-informed decisions about health care by preparing, maintaining, and promoting the accessibility of systematic reviews. These reviews are published electronically in The Cochrane Library, which is available on the Internet and CD-ROM. The Cochrane Collaboration was established in 1993 and is named after the British epidemiologist, Archie Cochrane.

## Updating Gould's Vision

As we progress through the 21st century, and health care information continues to become ever more plentiful, there are tremendous opportunities to make knowledge about health care more accessible. However, for this to happen without overwhelming the people who are trying to make health care decisions—for themselves or for someone else—the need for new research to be designed and reported using systematic reviews becomes ever more pressing.

Returning to George Gould's vision, but bringing it into the modern era, I hope for a system in which everyone making a decision about health care in any part of the world would be able, in 15 minutes, to obtain up-to-date, reliable evidence of the effects of interventions they might choose, based on all the relevant research. Journals, especially new ones such as *PLoS Medicine*, will help achieve this by only publishing a report of a new research study under the following conditions. First, the researchers must justify their study on the basis of a previous systematic review. Second, the journal should publish an updated systematic review (which incorporates the new study) within the new study, alongside it, or shortly thereafter.
